# The effects of hyperoxia on microvascular endothelial cell proliferation and production of vaso-active substances

**DOI:** 10.1186/s40635-017-0135-4

**Published:** 2017-04-13

**Authors:** Ilias Attaye, Yvo M. Smulders, Monique C. de Waard, Heleen M. Oudemans-van Straaten, Bob Smit, Michiel H. Van Wijhe, Rene J. Musters, Pieter Koolwijk, Angelique M. E. Spoelstra–de Man

**Affiliations:** 1 Department of Intensive Care, VU University Medical Center, Amsterdam, The Netherlands; 2 Department of Physiology, VU University Medical Center, Amsterdam, The Netherlands; 3 Department of Internal Medicine, VU University Medical Center, Amsterdam, The Netherlands

**Keywords:** Hyperoxia, Endothelial cells, In vitro, eNOS, ET-1, Peroxynitrite

## Abstract

**Background:**

Hyperoxia, an arterial oxygen pressure of more than 100 mmHg or 13% O_2_, frequently occurs in hospitalized patients due to administration of supplemental oxygen. Increasing evidence suggests that hyperoxia induces vasoconstriction in the systemic (micro)circulation, potentially affecting organ perfusion. This study addresses effects of hyperoxia on viability, proliferative capacity, and on pathways affecting vascular tone in cultured human microvascular endothelial cells (hMVEC).

**Methods:**

hMVEC of the systemic circulation were exposed to graded oxygen fractions of 20, 30, 50, and 95% O_2_ for 8, 24, and 72 h. These fractions correspond to 152, 228, 380, and 722 mmHg, respectively. Cell proliferation and viability was measured via a proliferation assay, peroxynitrite formation via anti-nitrotyrosine levels, endothelial nitric oxide synthase (eNOS), and endothelin-1 (ET-1) levels via q-PCR and western blot analysis.

**Results:**

Exposing hMVEC to 50 and 95% O_2_ for more than 24 h impaired cell viability and proliferation. Hyperoxia did not significantly affect nitrotyrosine levels, nor eNOS mRNA and protein levels, regardless of the exposure time or oxygen concentration used. Phosphorylation of eNOS at the serine 1177 (S1177) residue and ET-1 mRNA levels were also not significantly affected.

**Conclusions:**

Exposure of isolated human microvascular endothelial cells to marked hyperoxia for more than 24 h decreases cell viability and proliferation. Our results do not support a role of eNOS mRNA and protein or ET-1 mRNA in the potential vasoconstrictive effects of hyperoxia on isolated hMVEC.

**Electronic supplementary material:**

The online version of this article (doi:10.1186/s40635-017-0135-4) contains supplementary material, which is available to authorized users.

## Background

Supplemental oxygen (O_2_) is frequently administered in the hospital, especially in critically ill patients. For years, oxygen therapy focused on avoiding hypoxia, arterial oxygen levels below 70 mmHg or 9% O_2,_ often accepting a state of hyperoxia, arterial oxygen levels of more than 100 mmHg or 13% O_2_ [1]. The consequences of hyperoxia, however, remain unclear and have been a topic of debate for several decades in which both beneficial as well as deleterious effects have been reported [2–5].

Clinically, hyperoxia is associated with negative outcomes such as acute lung injury [6], increased infarct size in patients with a myocardial or cerebral infarction [7, 8] and increased mortality in patients after cardiac arrest [9, 10]. In contrast, others report beneficial effects with improved organ function after cardiac arrest [11] and antimicrobial activity with a reduction of surgical site infections [12, 13].

Furthermore, hyperoxia can cause significant hemodynamic alterations [2, 3] and has been reported to induce vasoconstriction in several (cerebral, coronary, skeletal muscle, and retinal) [14–17], but not all (renal, mesenteric) [18, 19], vascular beds. This vasoconstriction appears mainly to occur at microvascular level, since the diameter of large conduit arteries remains constant [15, 20]. It may lead to heterogeneity of the microcirculation with loss of functional capillary density and impaired organ perfusion [3, 21], but may also stabilize hemodynamics in vasodilatory shock [5]. In addition, other studies show that hyperoxia is able to redistribute blood flow which can protect hepatosplanchic organs and the kidneys [22, 23].

Crucial in the pathophysiology of a large variety of clinical conditions, such as sepsis, trauma, or ischemia/reperfusion injury is microvascular dysfunction. Hyperoxia is thought to increase reactive oxygen species (ROS) which can damage the endothelium and can worsen microvascular dysfunction with increased permeability, local inflammation and coagulation, and disturbed hemodynamics [24, 25]. It is therefore important to gain more insight in the effects of hyperoxia on the microvascular endothelium.

Several studies investigated the direct effects of hyperoxia on cultured endothelial cells with controversial results. Exposure to hyperoxia exerted a toxic effect with induction of cell death and reduced cellular proliferation in several cell types, such as human lung microvascular endothelial cells and bovine adrenal capillary endothelial cells [26, 27]. Exposure to hyperoxia also led to an inflammatory status by increasing the expression of inflammatory molecules, such as intercellular adhesion molecule 1 (ICAM-1) in human pulmonary artery endothelial cells and human umbilical vein endothelial cells [28]. Conversely, other studies showed that hyperoxia decreased the number of apoptotic cells and had anti-inflammatory effects in a rat intestinal ischemia-reperfusion and sepsis model [29, 30].

Increased formation of ROS also appear to be of pivotal importance with regard to the effects of hyperoxia on cell viability and vascular tone [25, 31–33].

The exact underlying mechanisms of vasoconstriction following hyperoxia however remain unclear. The majority of the studies indicate a central role for the endothelium [16, 34, 35]. Increased oxidative stress can augment the formation of peroxynitrite (ONOO^−^), which is formed when superoxide (O_2_^−^) reacts with nitric oxide (NO), resulting in a reduced bioavailability of NO [36]. NO itself is crucial for endothelium-driven vasodilation and vascular homeostasis [37]. It has also been hypothesized that hyperoxia decreases NO levels by affecting endothelial nitric oxide synthase (eNOS), the main enzyme responsible for the production of NO by the endothelium [34, 35]. Another potential pathway is a hyperoxia-induced decrease of cyclooxygenase (COX) activity in endothelial cells, thereby possibly lowering the levels of vasodilatory prostaglandins [38]. Interestingly enough the effects of hyperoxia on the protein endothelin-1 (ET-1), which is a potent vasoconstrictor, remain scarcely investigated [39].

Studies investigating the effects of hyperoxia on vascular smooth muscle cells (VSMC) showed that hyperoxia can induce vasoconstriction via L-type calcium and by influencing 20-hydroxyeicosatetraenoic acid (20-HETE) production [40, 41].

Unfortunately, most animal and in vitro studies only investigated extreme hyperoxia (i.e. 95% O_2_), often using endothelial cells from the retinal or pulmonary circulation [16, 33, 34, 38, 42–46]. Studies using endothelial cells of the systemic microcirculation and milder degrees of hyperoxia are scarce.

Taken together, several lines of evidence suggest that hyperoxia affects endothelial cell viability and proliferative capacity, but also promotes vasoconstriction primarily via the endothelium. In this study the effects of hyperoxia on isolated human microvascular endothelial cells (hMVEC) were investigated, using a clinically relevant scale of oxygen exposure. We investigated the effects of hyperoxia on cell viability and proliferation. We also addressed potential underlying mechanisms by which hyperoxia induces toxicity and vasoconstriction by studying peroxynitrite formation via protein tyrosine nitration capacity, the NO pathway via eNOS mRNA and protein levels and ET-1 mRNA production.

## Methods

### Cell culture

The study was executed in accordance with the Declaration of Helsinki and was approved by the University Human Subjects Committee of the VU University Medical Center. Written informed consent was obtained from all healthy donors in accordance with the institutional guidelines. All healthy foreskins were collected anonymously and were kindly provided by the Department of Dermatology (VUmc, Amsterdam). Human microvascular endothelial cells (hMVEC) were isolated from the foreskins and characterized, as previously described, by the presence of CD31, VE-cadherin, and Von Willebrand factor [47]. Cells were cultured on gelatin-coated wells (1% w/v) in EBM-2 medium supplemented with EGM-2 MV SingleQuots, 100 U/ml penicillin, 100 mg/ml streptomycin (p/s), 2 mM L-glutamine, 5 U/ml heparin (all from Lonza, Verviers, Belgium). The fetal calf serum (FCS) was replaced with 10% human platelet lysate (hPL) which was acquired as previously described [48]. Cells were cultured in an incubator kept at 37°C with 95% air, 5% CO_2_, and 95% humidity. Since the cells were cultured under 5% CO_2_, the oxygen exposure changed from atmospheric levels (21% O_2_) to 20% O_2_. Confluent cells were washed with 0.5 mM EDTA (Merck Milipore, MA, USA) in HBSS and were trypsinized with 0.05% trypsin in EDTA/HBSS (Lonza, Verviers, Belgium). At least three cell donors from passage 9–11 were used for all the experiments.

### Hyperoxia exposure

Cells of a single donor were cultured on four different culture plates. The plates were placed in airtight acryl boxes with moist tissues to maintain humidity. At the start of each experiment, the boxes were flushed for 5 min with the indicated oxygen concentration (Table 1) by using two outlets. The boxes were thereafter placed in an incubator and kept at 37 °C. Oxygen concentration in the boxes was measured at the start and end of each experiment (Additional file 1: Figure S1) using the Microx 4 (PreSens, Regensburg, Germany). Experiments were halted if the boxes proved not to be airtight, as determined by a drop in oxygen concentration.

**Table 1:** Oxygen scale used for the experiments

Oxygen (%)	Oxygen (mmHg)
20	152
30	228
50	380
95	722

### Proliferation assay

A proliferation assay was performed to determine the effects of hyperoxia on cell death and proliferative capacity. Endothelial cells were exposed to 20% (control), 50, and 95% O_2_ for 3 days. Exposure to 30% O_2_ was not performed in this assay, due to limitations in the number of airtight boxes. Furthermore, pilot experiments showed that exposure to 30% O_2_ did not affect cell death and proliferative capacity when compared to 20% O_2_ (control). Cells from three donors were pooled and seeded in duplicate with a density of 13 × 10^3^ cells/cm^2^ on 12 wells 1% w/v gelatin-coated culture plates. The plates were placed in airtight boxes and were exposed to the indicated oxygen concentration. Pictures were taken every 24 h using a phase-contrast microscope. Cells were counted using ImageJ version 1.49.

### Peroxynitrite

Formation of peroxynitrite (ONOO^−^) was measured indirectly using anti-nitrotyrosine antibodies as previously validated [49, 50]. For this assay hMVECs were cultured in 8-well immunofluorescence μ-slides (Ibidi, Planegg, Germany, 80826). The slides were coated with 1% w/v gelatin which was cross-linked for 15 min using 2% glutaraldehyde (Sigma-Aldrich, St. Louis, MO, USA) at 37 °C. Cells were exposed to the various oxygen concentrations in airtight boxes for 24 h. The cells were thereafter washed using phosphate-buffered saline (PBS), were fixed with 4% paraformaldehyde (PFA, Sigma-Aldrich) for 15 min, and were permeabilized using 0.2% triton X-100 (Merck Millipore) for 5 min. The cells were incubated with the primary polyclonal rabbit anti-human anti-nitrotyrosine antibody (Life Technologies, Bleiswijk, The Netherlands) diluted (1:75) in PBS with 0.1% albumin (PBS-A, Sigma-Aldrich) overnight at 4 °C. The following day, the slides were washed using PBS with 0.05% Tween (PBS-T, Sigma-Aldrich) for 5 min. The slides were then incubated for 1 h with Alexa Fluor 488 conjugated donkey anti-rabbit secondary antibody (Molecular Probes Europe, Leiden, The Netherlands) (1:50) in PBS-A. Afterwards, the slides were washed with PBS-T and were mounted using Ibidi Mounting Medium (Ibidi, Planegg, Germany).

The immunofluorescence was determined using a Zeiss Axiovert 200 M Marianas™ inverted microscope (Carl Zeiss, Jena, Germany). The microscope, camera, and data were controlled by SlideBook™ software (SlideBook™ (Intelligent Imaging Innovations, Denver, CO, USA). SlideBook software was also used to determine the mean fluorescence intensity (MFI) per cell.

### Endothelin-1 (ET-1)

The effects of hyperoxia at the ET-1 mRNA level and the EDN1 gene were investigated by using quantitative PCR (q-PCR). For this assay, total RNA was isolated using the RNeasy Micro kit according to the manufacturer’s protocol (Qiagen, Hilden, Germany). The RNA quality and concentration was determined by Nanodrop (ISOGEN Life science, De Meern, The Netherlands). 100 μl total copy DNA (cDNA) was synthesized from 1000 ng/ml RNA with the cloned AMV First-Strand cDNA synthesis kit (Invitrogen). Q-PCR was performed in duplicate using Sybr Green (MESA Green QPCR Mastermix Plus for Sybr Assay, Eurogentec, Seraing, Belgium) in an ABI7500 RT-PCR machine (Applied Biosystems, Foster City, USA). Primers were self-designed and synthesized by Invitrogen (Invitrogen, Carlsbad, USA) (Table 2). Primer specificity was tested by homology search with the human genome (BLAST) and was confirmed by dissociation curve analysis. Relative expression levels (*N*-fold differences) of target genes were calculated using the delta delta Ct method, as previously described [51].

**Table 2:** Nucleotide sequences of the primers used in the q-PCR experiments

Target mRNA	Target protein	Forward primer	Reverse primer
NOSIII	eNOS	TGGCTTTCCCTTCCAGTTC	AGAGGCGTTTTGCTCCTTC
EDN1	ET-1	TGTGTCTACTTCTGCCACCTG	TGGCTAGCACATTGGCATCTA
ACTB	Beta-actin	AACTCCATCATGAAGTGTGACG	GATCCACATCTGCTGGAAGG

*NOSIII/eNOS* endothelial nitric oxide synthase, *EDN1* preproendothelin-1, *ET-1* endothelin-1

### Endothelial nitric oxide synthase (eNOS)

NO is difficult to measure directly due to its instability. The effects of hyperoxia were therefore determined on eNOS, the key enzyme responsible for NO generation in endothelial cells. The effects were investigated at the mRNA level, using q-PCR as described above. Protein expression and phosphorylation status of the serine 1177 (S1177) residue were determined using western blot.

For the western blot assay cell lysates of hMVEC were prepared by scraping the wells on ice using a rubber policeman and by adding Roche lysis buffer with phosphatase and protease inhibitors (Roche, Mannheim, Germany). Protein concentrations were determined using a Bradford assay. The lysates were mixed with Laemmli sample buffer (Bio-Rad, Hercules, USA) with 5% β-mercaptoethanol and were boiled for 5 min at 95 °C. Sodium dodecyl sulfate polyacrylamide gel electrophoresis (SDS-PAGE) was performed using a 6% gel. Proteins were blotted onto nitrocellulose membranes and were blocked with 5% BSA in tris-buffered saline with 0.05% Tween (TBS-T) for 1 h. Membranes were probed with the primary rabbit polyclonal antibodies (1:1000 in 5% BSA in TBS-T; eNOS, ABCAM; peNOS S1177, Cell signaling; ACTN-1, Life Technologies) overnight at 4 °C. Blots were washed the following day and were probed with a secondary goat-anti rabbit horseradish peroxidase-conjugated antibody (1:2000 in 5% BSA in TBS-T; Dako, Glostrup, Denmark) for 90 min at room temperature. The bands were visualized with enhanced chemiluminescence (GE Healthcare) on a LAS3000 machine (FUJIFILM).

### Statistical analysis

Statistical analyses were performed by one-way ANOVA with a post hoc Bonferroni test. The results were graphed using GraphPad Prism 6.0 for windows (GraphPad Software Inc, La Jolla, CA, USA). *P* < 0.05 was considered significant. The results are given as mean ± standard deviation (SD). 20% O_2_ was used as a control, since the cells were isolated and cultured under 20% O_2_, and an experimental setup using lower and more physiological levels of oxygen exposure as a control is not feasible in an in vitro setup. All hyperoxic conditions (30, 50, and 95% O_2_) were compared to 20% O_2_.

## Results

### Hyperoxia and cell proliferation

**Fig. 1:**
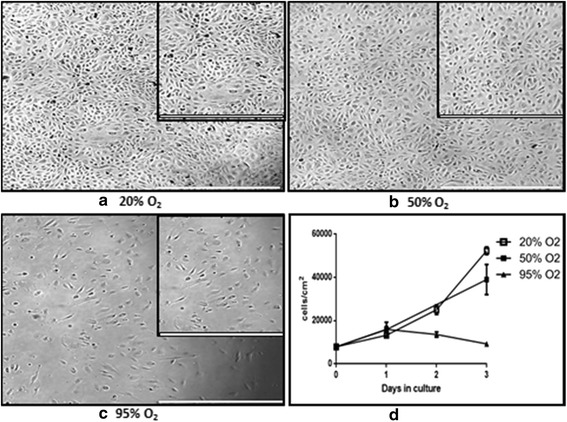
Effects of hyperoxia on cell proliferation. Pictures representing the status hMVEC after72 h of exposure to 20% O_2_ (control) **a**, 50% O_2_ **b**, and 95% O_2_ **c**. Proliferation displayed as cells/cm^2^ with mean of duplicates ± SD **d**. A pool of three different cell donors was used for the experiment, and the cells were seeded in duplicate. *White bars* represent 1000 μm

A proliferation assay was performed with a pool of three different cell donors to test if microvascular endothelial cells could withstand exposure to hyperoxia (Fig. 1). In this experiment, the wells were seeded in duplicate with 13 × 10^3^ cells/cm^2^. After initial seeding, not all cells adhered to the matrix, and the starting point was 7.9 × 10^3^ ± 0.2 × 10^3^ (mean ± SD) cells/cm^2^ for all conditions. Under 20% O_2_ (control) cells proliferated to 52 × 10^3^ ± 1.8 × 10^3^ cells/cm^2^ after 3 days, with a typical cobblestone morphology (Fig. 1a, Inset). Exposure to 50% O_2_ slightly reduced the proliferation to 39 × 10^3^ ± 7.0 × 10^3^ cells/cm^2^ at day 3 (Fig. 1d). The cells did however keep their cobblestone morphology after 72 h of exposure (Fig. 1b, Inset). Exposure to 95% O_2_ showed an initial increase in proliferation, which started to decrease after 24 h of exposure. This decrease became more pronounced as exposure time increased. Exposure to 95% O_2_ for 48 h resulted in 13.6 × 10^3^ ± 1.2 × 10^3^ cells/cm^2^. Exposure to 95% O_2_ for 72 h however showed a decrease to 9.3 × 10^3^ ± 0.2 × 10^3^ cells/cm^2^ indicating cell death. This was also confirmed by the observation of endothelial cells detaching from the matrix and floating within the cell culture media under the microscope. Exposure to 95% O_2_ also led to a more stressed morphology of cells, visible as stretched cells under the microscope. This was possibly due to lack of cell confluence following cell death and reduced proliferation (Fig. 1c, inset).

### Hyperoxia and peroxynitrite

**Fig. 2:**
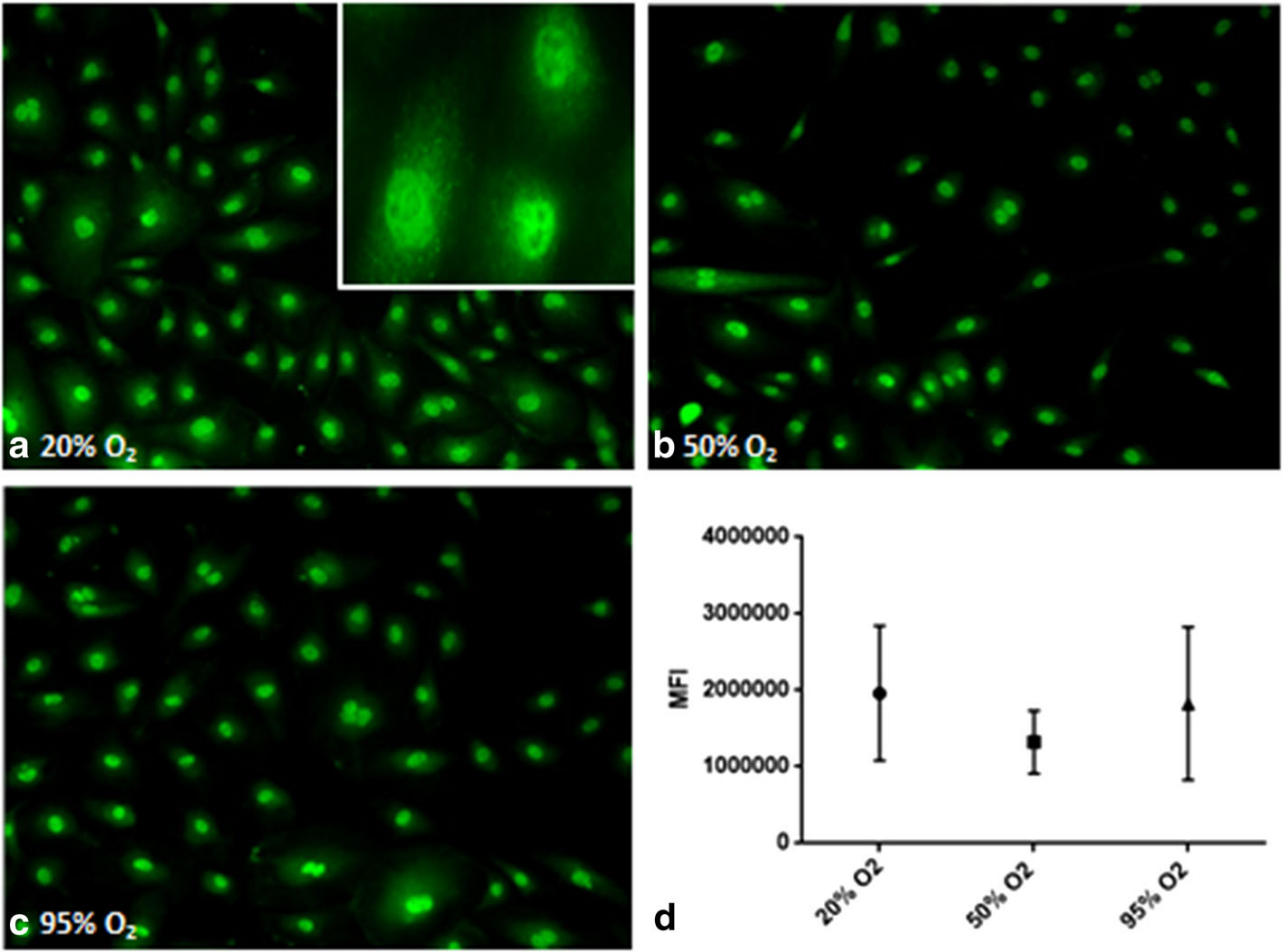
The effects of hyperoxia on nitrotyrosine levels in hMVEC. Figure shows representative pictures displaying **a** nitrotyrosine signal after 24 h exposure to 20% O_2_ (control) and localization of the signal under normal culture conditions, **b** nitrotyrosine signal after 24 h 50% O_2_ exposure, and **c** nitrotyrosine signal after 24 h 95% O_2_ exposure. **d** Mean fluorescence intensity (MFI) was calculated per cell, and data is expressed as mean ± SD; *N* = 3, all data non-significant (*P* > 0.05)

Immunofluorescence experiments were performed with three different cell donors in order to determine if hyperoxia affects the formation of peroxynitrite. Anti-nitrotyrosine antibodies were used for these experiments. Under normal culture conditions, the nitrotyrosine signal was observed predominantly in the proximity of the nuclei and cytoplasm of the fixated endothelial cells (Fig. 2a, inset). Exposure to 50 or 95% O_2_ for 24 h did not alter the nitrotyrosine localization (Fig. 2b, c). Exposure to hyperoxia did not increase nitrotyrosine levels in cultured hMVEC (Fig. 2d), indicating no rise in peroxynitrite levels, due to increased O_2_.

### Hyperoxia and eNOS

**Fig. 3:**
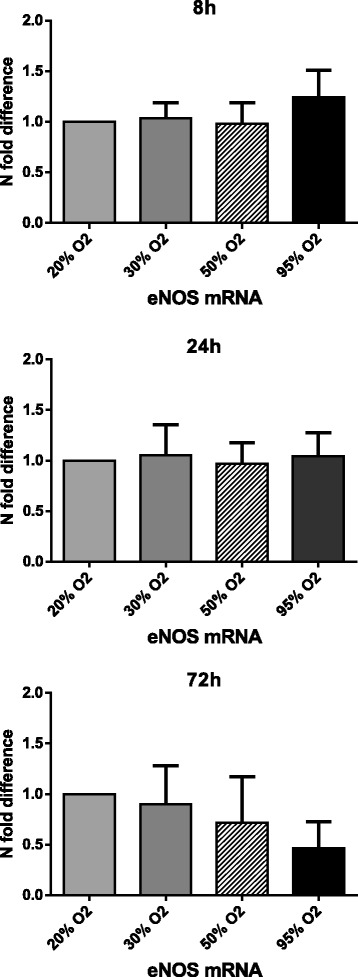
The effects of hyperoxia on eNOS mRNA expression. Gene expression of the eNOS gene was determined after 8, 24, and 72 h exposure under different oxygen concentrations. Data is expressed as *N*-fold difference with 20% O_2_ set as control (1.0). Mean ± SD; *N* = 4, all data non-significant (*P* > 0.05)

In order to determine if hyperoxia leads to vasoconstriction via a NO pathway, the effects were determined on both mRNA and protein levels of the enzyme eNOS, with at least three different cell donors. After 8, 24, and 72 h of exposure, eNOS mRNA levels did not change significantly compared to 20% O_2_ (control) (Fig. 3). Exposure to 95% O_2_ for 72 h did lead to a trend of decreased eNOS expression, which was not significant (*P* = 0.067).

No significant change was seen on eNOS protein levels after exposure to hyperoxia for 8, 24, and 72 h. Interestingly, an exposure time of 72 h gave an almost exact trend as an exposure time of 24 h. This trend is characterized by an initial increase followed by a decrease of the eNOS protein as oxygen concentrations increased.

The effects of hyperoxia were also determined with regards to the ratio of peNOS/eNOS, which is the ratio of the activated form of eNOS divided by the total eNOS protein.

**Fig. 4:**
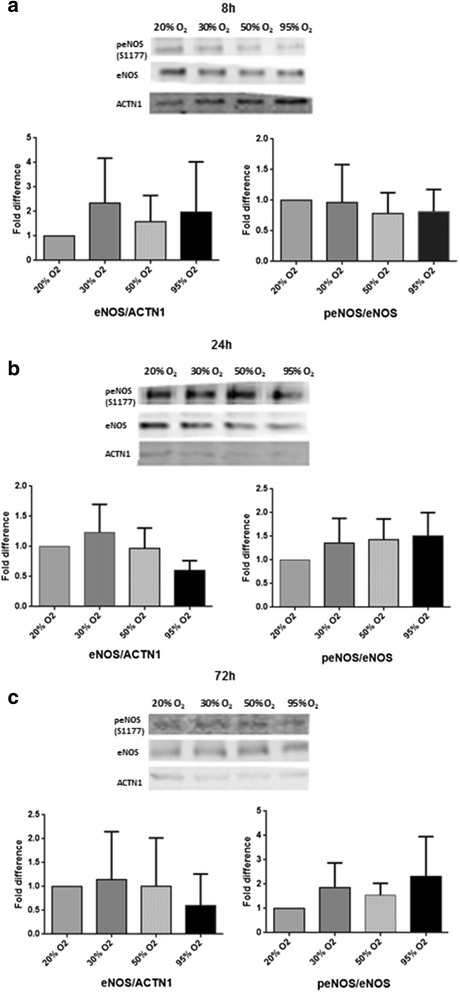
The effects of hyperoxia on total eNOS protein levels and on the peNOS/eNOS ratio. hMVEC were exposed to different oxygen concentrations for 8 (**a**), 24 (**b**), and 72 h (**c**). Phospho-eNOS (S1177), total eNOS, and α-actinine (ACTN1) protein levels were determined by western blotting. ACTN1 was used to correct for loading differences. Figure shows representative blots per time point of peNOS, eNOS, α-actinine, and the quantification of the western blot results using densitometry. 20% O_2_ is used as a control (set at 1.0). Data is expressed as mean ± SD; at least three different cell donors were used for the experiments. All data non-significant (*P* > 0.05)

Exposure to hyperoxia for 8 h gave a trend of a decreased expression of the ratio when exposed to different degrees of hyperoxia. Exposure for 24 and 72 h however gave a trend of increased expression of the ratio when exposed to different degrees of hyperoxia. These observed trends in the phosphorylation status were not statistically significant (Fig. 4).

### Hyperoxia and ET-1

The effects of hyperoxia on the mRNA levels of the potent vasoconstrictor ET-1 were also determined with four different cell donors.

**Fig. 5:**
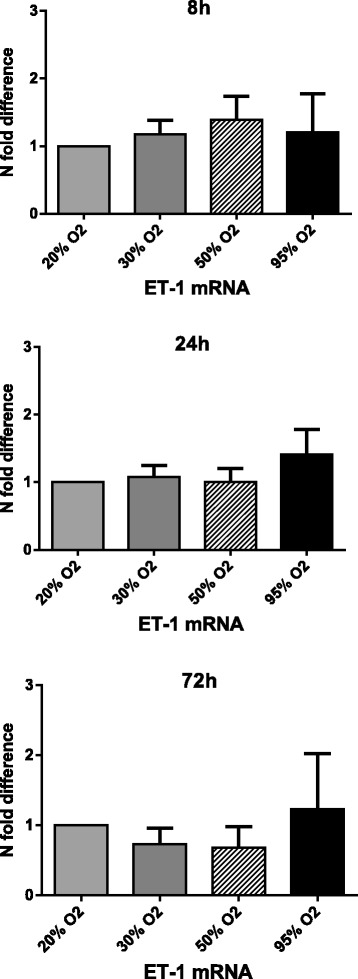
The effects of hyperoxia on ET-1 mRNA levels. hMVEC were exposed to different oxygen concentrations for 8, 24, and 72 h. Data is expressed as *N*-fold difference with 20% O_2_ set as control (1.0). Mean ± SD; *N* = 4, all data non-significant (*P* > 0.05)

Exposure to hyperoxia for 8 h led to a trend of increased expression of ET-1 mRNA when compared to 20% O_2_ (control). Exposure for 24 h led to an increase of 1.41 when the cells were exposed to 95% O_2_. Exposure for 72 h to 30 and 50% O_2_ led to a slightly decreased expression of 0.73 and 0.68 when compared to 20% O_2_ (control). Exposure to 95% O_2_ gave rise to a minor increase of 1.23 in the ET-1 mRNA expression. None of these changes were statistically significant (Fig. 5)

.

## Discussion

This cell culture study showed that exposure of isolated human systemic microvascular endothelial cells to 50% O_2_ for more than 24 h slightly affected cell proliferation, but exposure to 95% O_2_ for more than 24 h markedly reduced cell viability and proliferative capacity. Exposure to hyperoxia for 8, 24, and 72 h did not lead to a significant change in protein tyrosine nitration by peroxinitrite. Furthermore, exposure to hyperoxia did not significantly affect eNOS mRNA, protein, or serine-phosphorylation levels, nor did it alter the levels of ET-1 mRNA.

To the best of our knowledge, the current study is the first to investigate the role of hyperoxia on isolated cultured human endothelial cells of the systemic microcirculation using different degrees of hyperoxia. Systemic microvascular endothelial cells are particularly relevant, since in critically ill patients (who are most frequently exposed to hyperoxia during mechanical ventilation) microvascular dysfunction plays a pivotal role in conditions such as sepsis, trauma, and ischemia/reperfusion injury. An increase of microvascular endothelial injury, which is the largest endothelial surface of the body, may worsen organ failure.

Exposure to extreme hyperoxia (95% O_2_) in our study did not stop microvascular endothelial cell proliferation in the first day, but after 24 h, cell densities started to decrease which became more pronounced as exposure time increased. Exposure for 72 h decreased total cell numbers back towards seeding densities, indicating cell death. The toxic effects of extreme hyperoxia were also shown in a recent study which exposed human umbilical endothelial cells (HUVEC) to 95% O_2_ [52]. This study showed that the total number of cells remained equal after 8 h of exposure, whereas the number of apoptotic and dead cells had increased. After 72 h there was a 15% decrease in alive cells compared to 21% O_2_, which was used as a control. The toxic effects of extreme hyperoxia appear to differ between vascular beds. Pulmonary endothelial cells seem to be more resistant to the toxic effects of oxygen. In a study exposing isolated human microvascular endothelial cells of the lung to 95% O_2_, cell death was minimal until day 4 [27]. In another study, bovine pulmonary artery cells exposed to 95% O_2_ showed normal morphology, and no changes in cell number after 72 h compared to 21% O_2_, whereas bovine carotid artery endothelial cells developed irregular, atrophic, and distorted shapes after 48 h of exposure with a reduction to 63% of the control number of cells by 72 h [38].

With regard to moderate hyperoxia, in our study, exposure of systemic microvascular endothelial cells to 50% O_2_ for more than 24 h slightly reduced cell proliferation, but not viability. Similarly, a study of isolated bovine adrenal capillary endothelial cells reported that the proliferation was inhibited after 24 h of exposure to 40% O_2_. The cell number did not decrease in this study, but remained stable for the duration of the experiments, which was 6 days [26]. In a very recent study, HUVEC exposed to 40% O_2_ showed a significant reduction in viable cell count after 24 h [53]. In contrast, bovine carotid artery endothelial cells and bovine pulmonary artery endothelial cells exposed to 60% O_2_ did not show changes in morphology or cell number after 72 h of exposure [38].

Taken altogether, these results suggest that not only extreme hyperoxia, but also the clinically more relevant moderate hyperoxia may harm the microvascular endothelium. However, the response to extreme and moderate hyperoxic exposure can differ in endothelial cells from different vascular beds and different species [54]. In this in vitro study, we investigated whether the ROS peroxynitrite, measured by nitrotyrosine as an indirect marker, played a role in the toxic effects of hyperoxia. Exposure to hyperoxia did not significantly increase the nitrotyrosine signal within systemic microvascular endothelial cells. We hypothesize that exposure to hyperoxia might not lead to an increase in ROS via a peroxynitrite pathway in cultured endothelial cells. However, it is possible that basal NO levels were too low in this study to be able to increase the peroxynitrite levels adequately, since peroxynitrite formation is dependent on both O_2_^−^ as well as NO. This is in line with a previous study, which reported that hyperoxia only increases peroxynitrite formation after the addition of exogenous NO [27]. Our findings however do not exclude an increase in other ROS, as suggested by a study in rat pulmonary capillary endothelial cells [24]. In this study, exposure to hyperoxia increased oxidative stress, as was estimated by 2′,7′-dichlorofluorescein (DCF) which does not specify the type of ROS involved.

Our study investigated potential underlying mechanisms by which hyperoxia induces vasoconstriction. Exposure to extreme and moderate hyperoxia did not significantly affect eNOS mRNA, protein, or serine-phosphorylation levels, nor did it alter the levels of ET-1 mRNA. There are several explanations for these results.

First, it is possible that isolated endothelial cells are not a suitable model to study hyperoxic vasoconstriction. It could be that the O_2_ sensor is not located in the endothelium, but in other cells of the arteriolar wall (vascular smooth muscle cells), intraluminal (red blood cells), or in extravascular cells (parenchymal cells or mast cells). In addition, interaction with extravascular cells and smooth muscle cells may be necessary to activate the signaling pathway that couples changes in arteriolar oxygen pressure to changes in arteriolar tone [55].

Second, our in vitro model may not be suitable to investigate hyperoxic vasoconstriction, since NO availability is not guaranteed. In our study, no significant effect of hyperoxia on eNOS mRNA, protein levels, or serine-phosphorylation levels was found. However, the majority of studies in the literature point in the direction of a reduced bioavailability of NO as the underlying mechanism of hyperoxic vasoconstriction [32–35]. But for a model to show an effect on NO, the NO availability at the start of the experiment should be comparable to in vivo situations. The isolated endothelial cells were not exposed to flow, which may have decreased their basal NO level [56, 57].

Furthermore, it is possible that supporting leukocytes are needed in order to increase the NO levels. Hyperoxia can lead to an inflammatory status of the vascular system [28]. During this inflammation, NO production can be greatly increased by mainly macrophages, which possess the enzyme inducible nitric oxide synthase (iNOS). In the latter situation, an increase in peroxynitrite levels can be expected since both the NO as well as the O_2_^−^ will be increased [58]. These results could not be reproduced in our study, since iNOS regulation is largely controlled via macrophages and not endothelial cells [59].

However, another possible explanation for our negative results with regard to the NO pathway can be that hyperoxia does influence NO bioavailability, but that we were not able to detect it, since it is not possible to measure NO directly.

Third, another reason for the lack of effect of hyperoxia on eNOS and ET-1 in our study may be that hyperoxia induces vasoconstriction by affecting other vaso-active mediators. Several studies suggest a role for prostaglandins [16, 38] or 20-hydroxyeicosatetraenoic acid (20-HETE), a vasoconstrictor which is formed in vascular smooth muscle cells (VSMC) by the CYP450-4A enzyme system [40, 41, 43, 60]. In contrast with our ET-1 findings in human endothelial cells of the systemic microcirculation, hyperoxia did increase ET-1 levels in isolated bovine adrenal capillary endothelial cells and bovine retinal endothelial cells [61]. The difference in results can be based on species variability or upon different vascular origin of the endothelial cells used. Furthermore, we investigated ET-1 only at the mRNA and not the protein level, since general consensus within the literature states that the bioavailability of the ET-1 protein is predominately regulated at the transcriptional level of the EDN1 gene [39]. Therefore, we cannot exclude the possibility that ET-1 was affected at translation or posttranslational level.

Fourth, the negative results with regard to vasoconstrictive pathways may be caused by the use of 20% O_2_ as a control for cultured endothelial cells, because the cells were isolated and cultured under these conditions. Twenty percent of O_2_, however, is already hyperoxic in vivo, especially at tissue level [62]. This may have attenuated the differences between the groups.

Fifth, hyperoxia might not stimulate vasoconstrictive pathways in these specific microvascular endothelial cells derived from the foreskin, since the effect of hyperoxia on vascular tone varies between different vascular beds. For example, hyperoxia induced vasoconstriction in coronary arteries of pigs [34, 63] and in carotid vessels from dogs [64], but induced vasodilation in renal vessels of the dog [65], whereas no response to hyperoxia was observed in the arterioles of the mesentery of the rat and cat [19, 66].

More research is needed exploring the abovementioned pathways in an isolated and combined way in vitro as well as in vivo. Studies combining cultured endothelial cells, leukocytes, and vascular smooth muscle cells with flow and inflammatory stimuli can help to further unravel the mechanisms behind hyperoxic vasoconstriction.

### Limitations

Several limitations exist in this study. For all experiments, at least three different cell donors were used to correct for donor variability. Although this is not uncommon within in vitro literature, the power of our study is limited. Another possible limitation is the use of 20% O_2_ as a control for the experiments, because the endothelial cells were isolated and cultured under these conditions. 20% O_2_ is however already hyperoxic for endothelial cells in vivo [62]. However, performing experiments using physiological levels of oxygen exposure as a control would require endothelial cells to be isolated and cultured continuously within a hypoxic chamber. This is not feasible within an in vitro setup. Performing experiments using 20% O_2_ as a control is common practice and a limitation within in vitro literature in general. We did consider this point and repeated the eNOS and ET-1 experiments by exposing them to 10% O_2_ (=76 mmHg) (Additional file 1: Figure S2). This did not lead to significantly different outcomes of the experiments. A limitation of this study was the fact that the cells could only be cultured under hyperoxia for a maximum of 72 h. After 72 h, the culture media needed to be refreshed and the airtight boxes opened. This would decrease the oxygen concentration within the boxes back to atmospheric conditions. Experiments investigating hyperoxic exposure for short time periods (i.e., <1 h) were also not performed in this study. In addition, cell proliferation and viability experiments were not performed under mild hyperoxic (30% O_2_) conditions. Furthermore, this study investigated ET-1, via mRNA and eNOS, via mRNA and protein levels and did not investigate downstream pathways of NO generation or breakdown. Neither were other pathways investigated such as 20-HETE or prostaglandin production. Finally, our isolated hMVEC model precluded measuring hyperoxic effects mediated by the interaction between endothelial cells, leukocytes, and vascular smooth muscle cells and excluded the interaction with flow and circulating mediators.

## Conclusions

The present model of isolated and cultured human microvascular endothelial cells suggests that not only extreme hyperoxia (95% O_2_), but also the clinically more relevant moderate hyperoxia (50% O_2_) for more than 24 h may harm the microvascular endothelium. This is especially relevant for critically ill patients, where microvascular dysfunction is frequently present. Hyperoxia may worsen microvascular endothelial injury and may contribute to multiple organ failure. Because control experiments in vitro were done under hyperoxic conditions (20% O_2_) relative to the real tissue conditions in vivo, already beginning toxic effects in the controls (20% O_2_) cannot be excluded. Peroxynitrite did not seem to play a major role in the reduced viability and proliferation in vitro. Furthermore, in this model of isolated systemic human microvascular endothelial cells hyperoxia did not affect several key elements of the vaso-active response, namely, eNOS mRNA and protein and ET-1 mRNA levels. Hyperoxic vasoconstriction remains a complex mechanism, and it is possible that isolated in vitro models alone are not sufficient to study this mechanism. To show the full impact of endothelial responses to hyperoxia, models that allow the interaction between endothelial cells, leukocytes, and vascular smooth muscle cells in combination with flow and inflammatory stimuli are likely more appropriate.

## Additional file

Additional file 1: Figure S1. A representative example of oxygen stability during the experiments. The other oxygen percentages used had a similar pattern. **Figure S2.** The eNOS and the ET-1 experiments of Figs. 3, 4, and 5. displayed as *N*-fold difference, comparing 10% O_2_ exposure to 20% O_2_ (control, set as 1.0) and hyperoxia. Data is expressed mean ± SD; all data non-significant (*P* > 0.05). (DOCX 266 kb)

## Abbreviations

20-HETE: 20-hydroxyeicosatetraenoic acid
BSA: Bovine serum albumin
cDNA: Copy DNA
COX: Cyclooxygenase
eNOS: Endothelial nitric oxide synthase
ET-1: Endothelin-1
FCS: Fetal calf serum
hMVEC: Human Microvascular Endothelial Cells
hPL: Human platelet lysate
HUVEC: Human umbilical vein endothelial cells
ICAM-1: Intercellular adhesion molecule 1
iNOS: Inducible nitric oxide synthase
MFI: Mean fluorescence intensity
NO: Nitric oxide
O_2_: Oxygen
O_2_^−^: Superoxide radical
ONOO^−^: Peroxynitrite
PBS: Phosphate-buffered saline
peNOS: Phospho-eNOS
PFA: Paraformaldehyde
ROS: Reactive oxygen species
S1177: Serine 1177
SDS-PAGE: Sodium dodecyl sulfate polyacrylamide gel electrophoresis
TBS: Tris-buffered saline
