# Supplementary figures and images for: The effects of hyperoxia on microvascular endothelial cell proliferation and production of vaso-active substances
Source: Intensive Care Med Exp. 2017 Apr 13;5:22. doi: 10.1186/s40635-017-0135-4 (PMC5391371; doi:10.1186/s40635-017-0135-4)

**Supplementary figures manuscript**

**Supplementary figure S1**


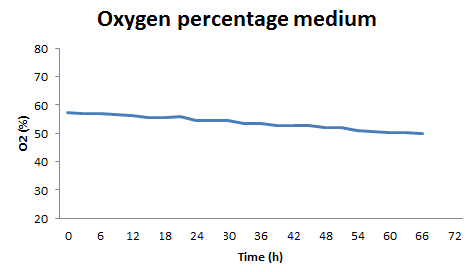


**Supplementary figure S2**


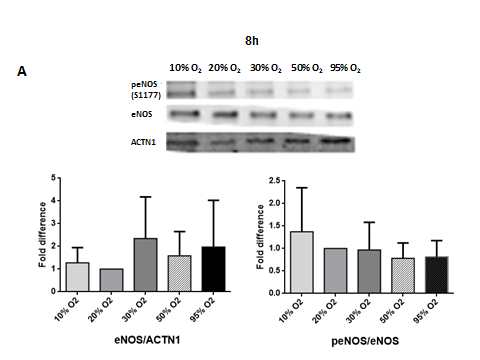


**24h**


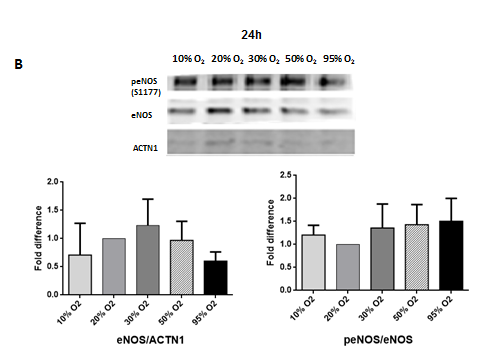


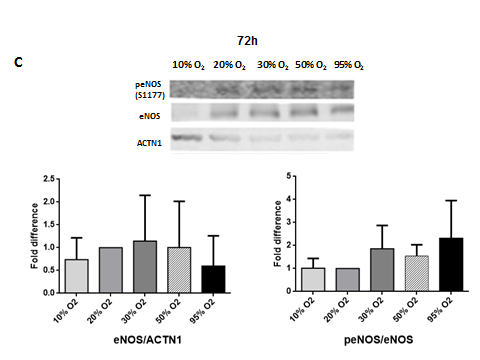

Supplement: Additional file 1: Figure S1. — A representative example of oxygen stability during the experiments. The other oxygen percentages used had a similar pattern. Figure S2. The eNOS and the ET-1 experiments of Figs. 3, 4, and 5. displayed as N-fold difference, comparing 10% O2 exposure to 20% O2 (control, set as 1.0) and hyperoxia. Data is expressed mean ± SD; all data non-significant (P > 0.05). (DOCX 266 kb) [file 40635_2017_135_MOESM1_ESM.docx]
